# Assessing the Prevalence of Doping Among Elite Athletes: An Analysis of Results Generated by the Single Sample Count Method Versus the Unrelated Question Method

**DOI:** 10.1186/s40798-023-00658-5

**Published:** 2023-11-28

**Authors:** Rolf Ulrich, Léa Cléret, R. Dawn Comstock, Gen Kanayama, Perikles Simon, Harrison G. Pope

**Affiliations:** 1 Department of Psychology, University of Tübingen, 72076 Tübingen, Germany; 2 Leadership Trust Training and Development, Upper Granary, Brockhampton, HR4 1SE UK; 3 Colorado School of Public Health, University of Colorado, Aurora, CO USA; 4 Biological Psychiatry Laboratory, McLean Hospital, 115 Mill St., Belmont, MA 02478 USA; 5 Department of Psychiatry, Harvard Medical School, Boston, MA USA; 6 Department of Sports Medicine, Rehabilitation and Disease Prevention, Johannes Gutenberg University Mainz, 55131 Mainz, Germany

**Keywords:** Doping, Randomized response technique, Unrelated question method, Single sample count, Elite athletes

## Abstract

In 2011, a group of researchers investigated the 12-month prevalence of doping at the 13th International Association of Athletics Federations World Championships in Athletics (WCA) in Daegu, South Korea, and also at the 12th Pan-Arab Games (PAG) in Doha, Qatar. The prevalence of doping at each event was estimated using an established randomized response method, the Unrelated Question Model (UQM). The study, published in 2018, found that the prevalence of past-year doping was at least 30% at WCA and 45% at PAG. At both events, separate data sets were collected in addition to the UQM data using a new method, the single sample count (SSC). Recently, Petróczi et al. have reported 12-month doping prevalence estimates for these two events based on the SSC data. These investigators obtained substantially lower prevalence estimates using the SSC and suggested that the 2018 estimates based on the UQM may have been too high. However, in this communication, we point out several possible shortcomings in the methods of Petróczi et al. and show that their SSC data would be equally compatible with a high 12-month doping prevalence comparable to the UQM estimates published in 2018.

## Key points

• A prior study of the prevalence of past-year doping and dietary supplement use among elite athletes, conducted at two international sporting events, used two randomized response techniques—the unrelated question method (UQM) and the single sample count (SSC)—to ensure the anonymity of the participants and thus encourage honest responses.
• The UQM analysis, published in 2018, found the prevalence of past-year doping to be at least 30% and 45% at the two events, respectively, and the prevalence of past-year dietary supplement use at the second event about 70%—a figure congruent with other studies of supplement use among elite athletes. However, the SSC analysis, published in 2022, yielded rates of only 21.2% and 10.6% for doping and only 8.6% for dietary supplements.
• In a reanalysis of the SSC data, using a slightly different model that considers both cheating and underreporting due to cognitive limitations, we show that the SSC data can yield much higher prevalence estimates, very similar to those of the UQM.

## Background

In 2011, the World Anti-Doping Agency (WADA) invited a group of researchers to develop survey methods for estimating the prevalence of doping behavior among elite athletes. After some pilot work, this group employed the well-established Unrelated Question Model (UQM) [1] to estimate the prevalence of past-year doping at two international sports events: the 13th International Association of Athletics Federations World Championships in Athletics (WCA) in Daegu, South Korea, and the 12th Pan-Arab Games (PAG) in Doha, Qatar, both held in 2011. The UQM method is described in detail in our prior publication emanating from this study [2], together with its supplemental material. Briefly, each athlete was presented with an initial question displayed on a tablet computer:

*Think of someone close to you (it can be anyone, such as your parent, sibling, partner, or even yourself) whose date of birth you know*.

The respondent is then directed to the next screen, which states as follows:

*Now think about the date of birth of the person you have chosen*.

*If the date is between the 1st and 10th day of a month, proceed to* ***Question A*** *and please answer it honestly.*

*If the date is between the 11th and 31st day of a month, proceed to* ***Question B*** *and please answer it honestly.*

The respondent then goes on to the next screen, which appears as follows:

***Question A:*** *Is the person’s date of birth in the first half of the year (January through June inclusive)?*

***Question B:*** *Have you knowingly violated anti-doping regulations by using a prohibited substance or method in the past 12 months?*

*Note that only you can know which of the two questions you are answering!*

It will be seen that this method guarantees the secrecy of each individual respondent’s answer, since the investigator cannot know the identity or the date of birth of the person chosen in the respondent’s mind. However, when assessing the total number of “yes” and “no” answers generated by a large sample of respondents, it is possible to compute the estimated number of dopers in the overall sample.

The prevalence estimates obtained with this UQM method were published in *Sports Medicine* in 2018 [2]. After performing several sensitivity analyses, the authors found that the prevalence of past-year doping was at least 30% at WCA and 45% at PAG. The authors also administered a UQM control question at PAG on the past-year use of dietary supplements. The estimated past-year supplement use at PAG was about 70%, a figure consistent with other studies of supplement use presented in a prior systematic review and meta-analysis [3].

At the two sporting events, a new method, the single sample count (SSC) [4], was tested and compared to the UQM. Dr. Andrea Petróczi, who had been the lead author of the original publication introducing the SSC method [4], was a member of the WADA prevalence group in 2011. She was interested in using the SSC in parallel with the UQM at the two elite athletic events in order to compare the estimates generated by the two methods. The full details of the SSC, together with an example, are presented below in the section of this paper entitled “critical issues.”

Unfortunately, the initial SSC doping prevalence estimates, based on the suggested procedure from Dr. Petróczi’s initial publication [4], were logically impossible values (i.e., less than zero)—casting doubt on the accuracy or reliability of the method. Accordingly, Petróczi and colleagues postponed publishing the SSC results pending further analysis. Consequently, only the raw data obtained using this method were included in the Supplementary Material of the 2018 publication, with an accompanying comment that the SSC estimates would be reported separately at a later date.

In collaboration with the members of the new WADA prevalence working group (Drs. Cruyff, de Hon, Sagoe, and Saugy), Dr. Petróczi has now published revised past-year SSC prevalence estimates for the two athletic events [5]. This analysis yielded estimates substantially lower than those previously obtained at the same events using the UQM, with values of 21.2% for doping at WCA, 10.6% for doping at PAG, and 8.6% for supplement use at PAG.

Moreover, in a further recent publication [6] which included Drs. Cruyff and de Hon as co-authors, it is stated:

*"A […] study on doping prevalence reported a prevalence of 43.6% (with a 95% confidence interval of 39.4–47.9%) among athletes at the International Association of Athletics Federations (IAAF) 2011 World Championships* (Ulrich et al. [2])*. An even higher prevalence of 57.1% (95% CI of 52.4–61.8%) was observed among athletes at the 12th Quadrennial Pan-Arab Games. However, it is important to note that the data of these two studies were recently critically reviewed and re-analysed, resulting in lower estimates of 21.2% and 10.6% respectively” (page 132).*

We would caution that the last sentence of this paragraph might lead readers to believe incorrectly that the investigators performed a re-analysis of the original raw UQM data and that this analysis resulted in lower estimates, suggesting that the original analysis was flawed. In fact, however, the investigators did not reanalyze the original UQM data; they simply compared their new SSC estimates with those previously published using the UQM.

## Critical Issues

Upon examining the 2022 article by Petróczi et al., several questions arise. First, the 8.6% estimate for the prevalence of dietary supplement use obtained with the SSC method appears unrealistically low, given that a systematic meta-analysis of supplement use among elite athletes, quoted above, reported average estimates of 69% and 71% for male and female athletes, respectively [3]. This striking difference suggests possible shortcomings in the SSC method as used here, and by extension raises questions regarding the estimates generated for doping at the two events, since these estimates were generated using the same methods.

Second, the authors suggest that non-compliance had biased the UQM prevalence estimates. This argument, however, is not particularly applicable to the UQM, but refers primarily to an entirely different randomized response technique (RRT), the “cheater detection model,” which can promote non-compliance [7]. With the cheater detection technique, a random device (e.g., the throw of a die) directs a respondent to honestly answer the sensitive yes/no-question (e.g., on doping) with probability *p* and to say “yes” on this same question with probability 1-*p* (a so-called forced yes-response). Consequently, the temptation to cheat is especially pronounced with the cheater detection model, because a respondent can eliminate any suggestion of being a doper by simply answering “no” when requested to say a forced “yes.” This temptation, however, appears much less likely to arise with the UQM technique, because the UQM does not require any forced-yes responses. We would also note the extensive sensitivity analyses provided by Ulrich et al. in their 2018 paper. These analyses, conducted to check the robustness of the UQM estimates using various scenarios of non-compliance, random guessing, etc., consistently revealed much higher prevalence estimates throughout multiple scenarios than did the SSC estimates in the 2022 paper.

Third, Petróczi et al. compared the UQM prevalence estimates to those obtained from other RRT studies of athletes, which usually reported lower prevalence estimates. Therefore, they again suggest that the 2018 UQM estimates may have been high. However, the sporting events in these other studies were typically regional or national events and hence less competitive than the international elite-level games of the WCA and PAG, which might well explain the lower prevalence of doping behavior reported in these other studies.

Fourth, the reported SSC data from the two sporting events are equally consistent with a high 12-month doping prevalence. We demonstrate this in the analysis below.

## Quantitative Modeling of the Observed SSC Data

In this section, we review Petróczi et al.'s method of analysis for the SSC data (which we will call the *P-model*). Then we introduce an alternative SSC model (which we call the *A-model*) that, in contrast to their model, yields a high 12-month doping prevalence and a much higher supplement prevalence than those produced by the P-model.

*SCC method*. In the SSC survey, participants are first asked to think of a person whose birth date the participant knows. Then the participant is presented with a list of five statements. For example, at PAG, the list read as follows:

1. *The birthday of the person I am thinking of falls in the second half of the year (July–December).*
2. *The birthday of the person I am thinking of is in February, April, June, August, October, or December.*
3. *The birthday of the person I am thinking of falls in the first half of the month (1–15 inclusively).*
4. *The birthday of the person I am thinking of is on an odd day (on or ending with 1, 3, 5, 7, 9).*
5. *I have knowingly violated anti-doping regulations by using a prohibited substance or method in the past 12 months.*

For each statement, participants were asked to determine whether it was true or not. For example, if a participant was thinking of an individual born on December 26, 1947, then the answers to questions 1 and 2 would be “true,” and the answers to questions 3 and 4 would be “false.” Therefore, if this participant were a doper he or she would have a total of 3 “trues,” whereas if he or she were not a doper than the number of “trues” would be 2. Importantly, however, to ensure the anonymity of the survey, participants could not manually mark each true statement as they went along, but were required to remember each “true” answer as they went along and mentally total them up at the end. Thus, respondents might have been at risk for undercounting the total number of “trues” since they did not have tick marks to refer back to. Furthermore, while carrying this mental sum in mind, participants then needed to navigate to a subsequent screen, where they were asked to press one of four response buttons to indicate whether “0 or 5,” "1,” "2,” “3,” or “4” of these five statements were true. The “0 or 5” category was introduced to prevent dopers from potentially exposing themselves as having answered yes to all innocuous questions. Table 1 contains the total number of observed responses for each response category and each SSC survey.^1^

**Table 1:** Number of SSC responses as a function of response category and survey

	“0 or 5”	“1”	“2”	“3”	“4”	M
WCA doping	154	333	426	185	105	1.796
PAG doping	77	274	308	212	83	1.948
PAG supplements	76	270	342	182	84	1.925

The last column gives the average number of responses (with the “0 or 5” option coded as zero true statements to calculate the average number under the assumption that the doping prevalence would be zero.)

*P-model* This model assumes that an innocuous statement is true with probability 1/2 and that the sensitive behavior is present with probability $\uppi$. Additionally, the model assumes that a participant is either non-compliant with probability $n$ or compliant with the complementary probability $1-n$. In the case of non-compliance, the participant randomly chooses a response from “0–5,” “1,” or “2.” By contrast, compliant participants honestly report the proper number of mentally counted true statements.

**Fig. 1:**
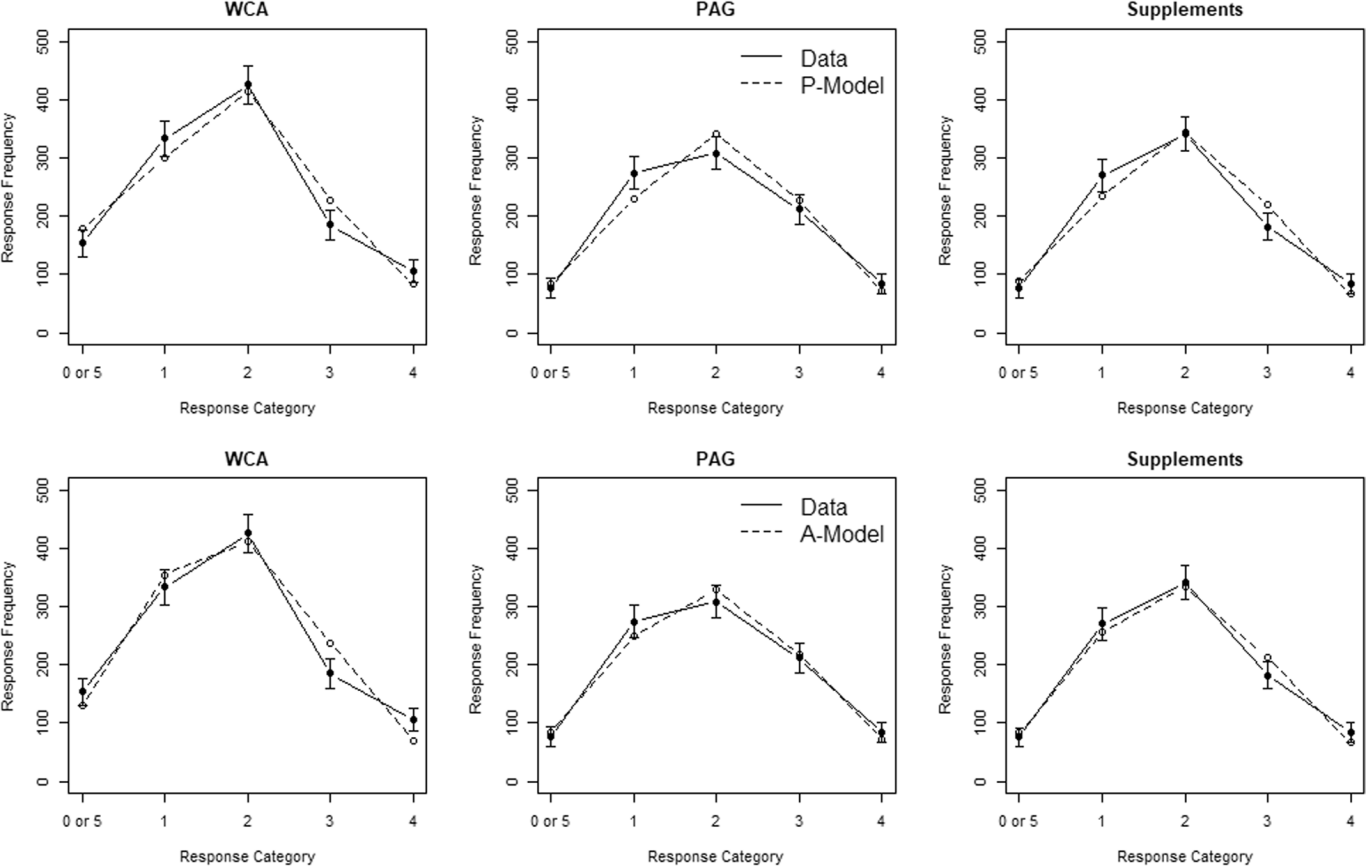
Predicted and observed response frequencies as a function of response category for each of the three surveys. The upper row of panels depicts the prediction for the P-model and the lower row for the A-model. Error bars represent 95% confidence intervals

We implemented the P-model using R software [8] and successfully reproduced the prevalence estimates reported by Petróczi et al. [5].^2^ The computer program minimized the ${G}^{2}$ statistics between observed and expected response frequency with a numerical search for the best fitting parameter combination (R routine *optim*). Table 2 (left side) contains the P-model fit results, and Fig. 1 (upper panels) shows the observed and expected frequencies for all three surveys.^3^

**Table 2:** Best model fits ${\mathrm{G}}^{2}$ for the P-model and the A-model along with the estimated model parameters $\widehat{\pi }$ (doping prevalence), $\widehat{\mathrm{n}}$ (non-compliance), and $\widehat{\mathrm{p}}$ (inclusion probability)

	P-model			A-model		
	${G}^{2}$	$\widehat{\pi }$	$\widehat{n}$	${G}^{2}$	$\widehat{\pi }$	$\widehat{p}$
WCA doping	20.6	21.2%	31.9%	35.0	36.7%	73.4%
PAG doping	15.5	10.6%	9.9%	6.3	39.9%	79.7%
PAG supplements	18.7	8.6%	11.4%	10.6	55.3%	70.5%
*Sum*	54.9	–	–	51.9

*A-model.* As an alternative to the P-model, we propose here the A-model. The main difference between the two models is as follows: The P-model assumes that non-compliance is due to cheating and therefore takes cheating into account, as described above. The A-model, however, accounts for both cheating and cognitive limitations. In other words, the A-model allows for two possible mechanisms by which participants may underreport the number of true statements. First, participants might intentionally fail to count every true statement in order to keep the total count strategically low—a “cheating” strategy similar to the P-model. Second, the A-model also allows that participants might accidentally undercount the number of true statements because of the cognitive burden of remembering the total number of true responses as they go along. Specifically, since participants were not able to place tick marks, and because capacity in working memory is limited [9], and even further reduced under stressful conditions [10],^4^ they might miss or forget to count a true response while mentally attempting to add up the total number of “trues.” Thus, each true statement enters the final count with a probability of $0.5\cdot p$ instead of $0.5$, with $p$ representing the probability that a given true statement is counted after allowing for both deliberate and accidental undercounting. Participants in the overall group answered “yes” to the doping question with probability $\uppi$. Therefore, $\uppi$ denotes a lower limit for the prevalence of doping.^5^

As with the P-model, we fitted the A-model to the observed response frequencies. Table 2 (right side) contains the estimated parameters and the goodness of fit, and Fig. 1 (lower panels) shows the observed and expected frequencies for the three surveys. It can be seen that this model yields much higher prevalence estimates than the P-model. Notably, the prevalence limits of this A-model align better than those of the P-model with the UQM estimates reported by Ulrich et al. [2]. As noted above, the prevalence estimates for the A-model in Table 2 are lower limits. If doping were underreported, similar to the innocent factual questions (e.g., $c = 0.75$), the prevalence estimates corrected for underreporting according to the A-model would be 48.9% for WCA, 53.2% for PAG, and 73.3% for supplements. These corrected values for underreporting doping correspond closely with the high UQM estimates of 43.6% (WCA), 57.1% (PAG), and 70.1% (supplements). Of particular note, the A-model yields a prevalence estimate for supplements that accords closely with other studies of supplement use among elite athletes [3], whereas the P-model yields an estimate of only 8.6% for supplements, as mentioned earlier.

It can be shown that the P-model may greatly underestimate the true doping prevalence if its assumptions do not hold. To demonstrate this phenomenon, we computed expected SSC frequency data for a hypothetical sample size $N=1000$ participants following the assumptions of the A-model with parameters $\uppi =0.55$ and $p=0.75$ (Table 3). The P-model was then fitted to these hypothetical frequency data. It can be seen in Table 3 that the best-fitting data of the P-model closely resemble the hypothetical data (${G}^{2}=1.2$). More crucially, the estimated prevalence $\widehat{\pi }=11.2\%$ from the P-model in this hypothetical sample would greatly underestimate the true underlying doping prevalence of 55%. In summary, a good model fit in this situation does not establish that the prevalence estimate is accurate (for a general discussion about this issue, see [11]).

**Table 3:** Hypothetical data according to the A-model and best-fitting data of the P-model (rounded values)

	“0 or 5”	“1”	“2”	“3”	“4”
Generated data according to A-model	80	249	350	241	81
Best-fitting predictions of the P-model	82	237	359	245	77

## Conclusion

In a study performed at two major international sporting events, the investigators used two randomized response techniques—the unrelated question method (UQM) and the single sample count (SSC)—to estimate the prevalence of doping and the prevalence of dietary supplement use among elite athletes. The UQM estimates, published in 2018 [2], suggested a past-year doping prevalence of at least 30% at one event and 45% at the other. By contrast, the SSC estimates published in 2022 yielded much lower estimates of 21.2% and 10.6% at the two events, respectively. An even greater divergence emerged on past-year supplement use, with the UQM yielding an estimate of about 70% (a figure consistent with prior studies of supplement use among elite athletes [3]), whereas the SSC yielded only 8.6% at the same event.

Does the UQM yield estimates that are too high or are the SSC estimates too low? In this commentary, we suggest that the analytic model used in the 2022 SSC paper (which we have termed the “P-model”) may underestimate the true prevalence of doping, and we show that the SSC data are consistent with a much higher prevalence of doping when using a plausible alternative model (which we have termed the “A-model”). In particular, the A-model yields a much more realistic estimate on the control question regarding the prevalence of supplement use than does the P-model. We also present a hypothetical scenario of 1000 athletes with a 55% prevalence of doping, where athletes report 75% of the actual number of “true” statements. In this scenario, the P-model would yield only an 11.2% estimated prevalence of doping, rather than the actual number of 55%—again suggesting that this model may not be the best for analyzing SSC data. At this point, therefore, we would suggest that methods for analyzing SSC data may deserve further refinement, and that the 2018 UQM results remain at this point the most plausible estimates of the frequency of past-year doping among elite athletes.

We would note that the analysis in this paper is focused on the methodological properties of the UQM versus the SSC, and does not speak to the prevalence of doping today, some 13 years after our original study was conducted. It would be of interest to conduct a similar study at current international athletic events, using the UQM, to assess whether doping remains as prevalent as we estimated in 2011 or whether increased attention to doping has reduced its prevalence in contemporary competitions.

## Data Availability

The frequency distribution of participants’ responses is provided in Table 1 of the paper, and the R code used to perform the analysis can be accessed at https://osf.io/crez2/?view_only=13f1a2d1125b4e69b24102f9fa3b1709 as noted in footnote 2.

## Abbreviations

WADA: World Anti-Doping Agency
UQM: Unrelated question method
WCA: World Championships in Athletics
PAG: Pan-Arab Games
SSC: Single sample count
IAAF: International Association of Athletics Federations
RRT: Randomized response technique

## Footnotes

1 The mean expected number of true innocuous statements should be 2. Thus, if the average number of all five statements is larger than 2, this would indicate that some participants have admitted to doping (e.g., Statement 5 above). According to this conjecture, doping prevalence could be estimated with $\widehat{\pi } = M - 2$, where $\widehat{\pi }$ denotes the prevalence estimate [4]. Unfortunately, as mentioned in the text above, this straightforward approach yields negative prevalence estimates. Given the failure of this approach, additional assumptions about the response behavior were required to correct these figures. This led Dr. Petrózci and colleagues to modifications of the original SSC model [12].

2 The R code for both the P-model and the A-model can be downloaded at https://osf.io/crez2/?view_only=13f1a2d1125b4e69b24102f9fa3b1709.

3 Note that a model parameter with a cap, such as $\widehat{\pi},$ denotes the estimate of $\pi$. This estimated parameter is based on fitting the model predictions to the SSC data.

4 This view is supported by the survey at WCA, where participants could indicate which of the two survey methods they preferred (SSC or UQM). 769 participants preferred UQM and 434 SSC, which probably indicates that UQM is less memory-demanding.

5 If dopers report doping in this SSC survey with probability $c$, then $\uppi =c\cdot {\uppi }_{T}$ applies, where ${\uppi }_{T}$ denotes the true doping prevalence. However, the SSC data only allow estimation of the lower prevalence limit $\uppi$ but not $c$ or ${\uppi }_{T}$. Nonetheless, with reasonable assumptions about $c$, the true prevalence could be inferred by referring to the above equation $\uppi =c\cdot {\uppi }_{T}$.

## References

[1] Greenberg BG, Abul-Ela A-LA, Simmons WR, Horvitz DG (1969). The unrelated question randomized response model: theoretical framework. J Am Stat Assoc.

[2] Ulrich R, Pope HG, Cléret L, Petróczi A, Nepusz T, Schaffer J (2018). Doping in two elite athletics competitions assessed by randomized-response surveys. Sports Med.

[3] Knapik JJ, Steelman RA, Hoedebecke SS, Austin KG, Farina EK, Lieberman HR (2016). Prevalence of dietary supplement use by athletes: systematic review and meta-analysis. Sports Med.

[4] Petróczi A, Nepusz T, Cross P, Taft H, Shah S, Deshmukh N (2011). New non-randomised model to assess the prevalence of discriminating behaviour: a pilot study on mephedrone. Subst Abus Treat Prev Policy..

[5] Petróczi A, Cruyff M, de Hon O, Sagoe D, Saugy M (2022). Hidden figures: Revisiting doping prevalence estimates previously reported for two major international sport events in the context of further empirical evidence and the extant literature. Front Sport Act Living.

[6] Balk L, Dopheide M, Cruyff M, Erik D, de Hon O (2023). Doping prevalence and attitudes towards doping in Dutch elite sports. Sci J Sport Perform.

[7] Clark SJ, Desharnais RA (1998). Honest answers to embarrassing questions: Detecting cheating in the randomized response model. Psychol Methods.

[8] Team R Core. R: A language and enviroment for statistical computing. R Foundation for Statistical Computing, Vienna, Austria. 2018.

[9] Cowan N (2001). The magical number 4 in short-term memory: a reconsideration of mental storage capacity. Behav Brain Sci.

[10] Eysenck MW, Calvo MG (1992). Anxiety and performance: the processing efficiency theory. Cognition Emotion.

[11] Roberts S, Pashler H (2000). How persuasive is a good fit? A comment on theory testing. Psychol Rev.

[12] Nepusz T, Petróczi A, Naughton DP, Epton T, Norman P (2014). Estimating the prevalence of socially sensitive behaviors: attributing guilty and innocent non-compliance with the single sample count method. Psychol Methods.

